# Supplementary material for: Transcriptional Responses for Biosynthesis of Triterpenoids in Exogenous Inducers Treated Inonotus Hispidus Using RNA-Seq
Source: Molecules. 2022 Dec 4;27(23):8541. doi: 10.3390/molecules27238541 (PMC9739630; doi:10.3390/molecules27238541)
Supplement: Supplementary file 1 [file molecules-27-08541-s001.zip › Table S3 ;The names and versions of all software used for RNA-seq.pdf]

Software List

Tools	Version	Description	Linkages
Trinity	v2.5.1	A Full-length transcriptome assembly tool from RNA-Seq data without a reference genome	https://github.com/trinityrnaseq/ trinityrnaseq/wiki
TransDecoder	v5.0.0	An tool for identifying candidate coding regions within transcript sequences	http://sourceforge.net/projects/transdecoder/
MISA	v1.0	A MIcroSAtellite identification tool	http://pgrc.ipk-gatersleben.de/misa/misa.html
BLAST	v2.2.31	Basic Local Alignment Search Tool	http://blast.ncbi.nlm.nih.gov/Blast.cgi
KOBAS2.0	v2.0	A software to identify statistically significantly enriched pathways using hypergeometric test	http://kobas.cbi.pku.edu.cn/help.do
HMMER	v3.1b	A tool for	http://hmmer.org/

	2	searching sequence databases for sequence homologs, and for making sequence alignments	
RSEM	v1.2.19	An accurate tool for quantifying transcript abundances from RNA-Seq data	http://deweylab.github.io/RSEM/
TGICL	v2.1	A software system for fast clustering of large EST datasets	http://www.tigr.org/tdb/tgi/software/
cd-hit-est	v4.6.1	A very widely used program for clustering and comparing protein or nucleotide sequences.	http://weizhongli-lab.org/cd-hit/
Blast2GO	v2.5	A bioinformatics platform for high-quality protein function prediction and	https://www.blast2go.com/

		functional analysis of genomic datasets.	
STAR	v2.6.0b	An ultrafast universal RNA-seq aligner	https://github.com/alexdobin/STAR
GATK	v3.2.2	A wide variety of tools with a primary focus on variant discovery and genotyping	https://software.broadinstitute.org/gatk/
DESeq2	v1.6.3	An R package for RNA-Seq Differential Expression Analysis based on a model using the negative binomial distribution	http://www.bioconductor.org/packages/release/bioc/html/DESeq.html
edgeR	v3.8.6	edgeR: a Bioconductor package for differential expression analysis of digital gene expression data	https://www.ncbi.nlm.nih.gov/pmc/articles/PMC2796818/
topGO	v2.28.0	An R package for gene ontology	http://www.bioconductor.org/packages/release/bioc/html/topGO.html

		enrichment analysis	
Cytoscape	v3.6.1	An open source software platform for visualizing complex networks	http://www.cytoscape.org/
